# Closure of the Fontanelles

**Published:** 1860-05

**Authors:** 


### CLOSURE OF THE FONTANELLES.

Physicians are often questioned about the proper time for the closure of the anterior fotanelle, and it may be difficult for some to answer, since the best anatomist are at variance on this point. We therefore think that it may not be unacceptable to give a summary of some recent observations, by Henri Roger, in the *Union Medicale* for November, 1859.

The researches are based upon the fact, that a cephalic souffle is not heard when the opening is closed by bone.

In three hundred children the anterior fontanelle was never found closed before the age of fifteen months, and never opened after the age of three years.

It must be stated, however, that a distinction is to be made between the clinical and anatomical closure—the first being recognizable during life, the second after death.

In the first case, that is the clinical closure, the size of the closure gradually diminishes, while, at the same time, the membrane becomes thicker until it finally feels like bone. When this takes place, the cephalic souffle is no longer perceptible. The only method of determining the absolute closure by bone, is to examine the dead body. Still, we may assume, that when the fontanelles appear to be closed by ossification, they really are so.

The results arrived at in the manner above mentioned, are as follows: The period of ossification is comprised between the ages of fifteen months and three years and a half. At the first age, the complete change is very rare; at the last, is always found. But these are the extremes. The occlusion generally takes place between the second and third year, and its frequency is regularly progressive from the twentieth to the twenty-third month, increases rapidly after the second year, and still constantly augments until the age of three and a half years.

Two diseases retard this change—rickets and hydrocephalus; the first by its influence upon the ossific process, the second by its mechanical action. The non-closure of the fontanelles at the usual time may be one of the first manifestations of rickets, and warn us of the approach of the disease.—*Boston Med. and Surg. Journal.*

